# Dentigerous cyst with parietal and intracystic calcifications: A case report and literature review

**DOI:** 10.4317/jced.54505

**Published:** 2018-03-01

**Authors:** Jordi Borrás-Ferreres, Alba Sánchez-Torres, José-Manuel Aguirre-Urizar, Cosme Gay-Escoda

**Affiliations:** 1 DDS. Fellow of the Master’s Degree Program in Oral Surgery and Orofacial Implantology (EHFRE International University/FUCSO); 2 DDS, MS. Master of Oral Surgery and Implantology. Associate Professor of Oral Surgery, School of Medicine and Health Sciences, University of Barcelona. Researcher at the IDIBELL Institute. Barcelona, Spain; 3 MD, DDS, PhD. Oral Medicine and Oral and Maxillofacial Pathology Unit. Dental Clinic Service. Master in Oral Pathology. Department of Stomatology II. UFI 11/25. University of the Basque Country / EHU. Leioa. Spain; 4 MD, DDS, MS, PhD, EBOS, OMFS. Chairman and Professor of the Oral and Maxillofacial Surgery Department, School of Medicine and Health Sciences, University of Barcelona. Director of Master’s Degree Program in Oral Surgery and Implantology (EHFRE International University/ FUCSO). Coordinator/Researcher at the IDIBELL Institute. Head of Oral and Maxillofacial Surgery and Implantology Department of the Teknon Medical Centre, Barcelona, Spain

## Abstract

**Background:**

Dentigerous cyst appears surrounding the crown of an included tooth. On the radiographic exam, a radiolucent rounded well-defined lesion can be observed.

**Material and Methods:**

This study reports a clinical case of a 34-years old man with a pericoronal radiolucent lesion associated to an impacted lower third molar with the presence of radiopaque material inside. The radiological differential diagnosis was calcifying odontogenic tumor, adenomatoid odontogenic tumor and dentigerous cyst. The impacted third molar was removed and the lesion was sent for the histopathological exam.

**Results:**

The histopathological diagnosis was dentigerous cyst with capsular calcifications. Specifically, parietal calcifications on its connective wall and a piece of cemento-osseous tissue inside.

**Conclusions:**

Non-neoplastic lesions such as dentigerous cysts could develop radiopacities inside the radiolucent pericoronal area.

** Key words:**Dentigerous cyst, calcifications, third molar, differential diagnosis.

## Introduction

Dentigerous cyst is a developmental odontogenic benign lesion which surrounds the crown of an included tooth (1). Radiographically, it appears as a well-defined unilocular radiolucent area (2). It currently represents around a 23% from all odontogenic cysts (3) and usually appears in men, at the third decade of life and mostly associated to included lower third molars up to 45% of cases (1). The cyst development is produced by an expansion of the reduced enamel epithelium due to liquid accumulation between the crown of the tooth and the epithelial components from the dental follicle (4). As observed on the histopathological exam, it has a connective cystic wall generally lined by a thin non-keratinized stratified epithelium (1).

Differential diagnosis has to include other pericoronal odontogenic lesions such as odontogenic keratocyst, unicystic ameloblastoma or adenomatoid odontogenic tumor (1,2). The treatment of choice is the surgical excision. The lesions have a good prognosis exempt from recurrences (5).

The aim of this study was to report and discuss a particular dentigerous cyst with calcifications on its connective wall.

## Case Report

**Figure 1:**
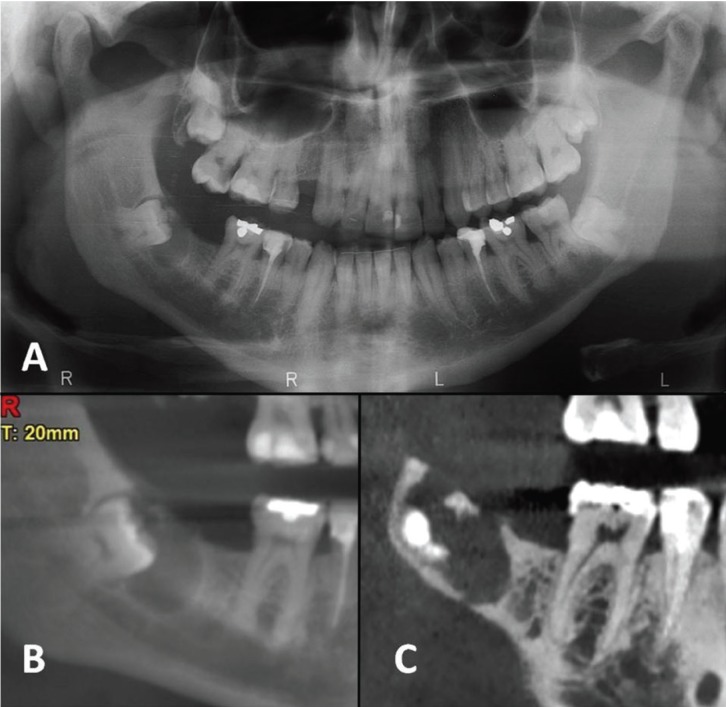
A) Ortopantomography shows a radiolucent pericoronal lesion associated to the right lower third molar with a radiopaque material inside. B) Panoramic slice from computed tomography (CT). Note the distal bone sclerosis and calcifications. C) Sagittal slice from CT shows the alveolar ridge resorption.

A 34-years old man, without any relevant medical background, attended to the Dental Clinic to perform a routine check-up. The orthopantomography revealed an included right lower third molar associated to a pericoronal radiolucency with a radiopaque area inside (Fig. 1A). Interestingly, a periapical radiograph from this area taken 10 years ago showed no lesion. A cyst or a benign odontogenic lesion was suspected. A computed tomography scan (CT-scan) displayed an area of bone resorption on the alveolar ridge, a thinning of the cortical bone, bone sclerosis on the distal area and the presence of a small radiopaque piece near to the occlusal aspect of the third molar (Fig. 1B,C). The radiological differential diagnosis was calcifying odontogenic tumor, adenomatoid odontogenic tumor and dentigerous cyst.

The surgical intervention was made under local anesthesia (4% articaine and 1:200.000 epinephrine) by a direct troncular technique to anesthetize inferior alveolar and lingual nerves, and an anesthetic infiltration on the buccal side and the retromolar mucosa for the buccal nerve. A triangular mucoperiosteal flap was elevated and then, the soft-consistency lesion fenestrating the alveolar ridge was observed. Ostectomy was performed with a number 8 round tungsten carbide bur for hand piece under saline solution irrigation. Then, the complete enucleation of the lesion was done and a turbid liquid and a small piece of hard tissue emerged from the inside. Subsequently, the extraction of the third molar was made maintaining the integrity of the neurovascular bundle of inferior alveolar nerve located lingually. Finally, the wound was sutured with 3/0 silk. The patient did not present intraoperative nor postoperative complications until a follow-up period of 2 years.

The lesion was immersed in a 10% formaldehyde solution and sent to the Oral and Maxillofacial Pathology and Diagnosis Service (SDPOMF) for the histopathological exam.

**Figure 2:**
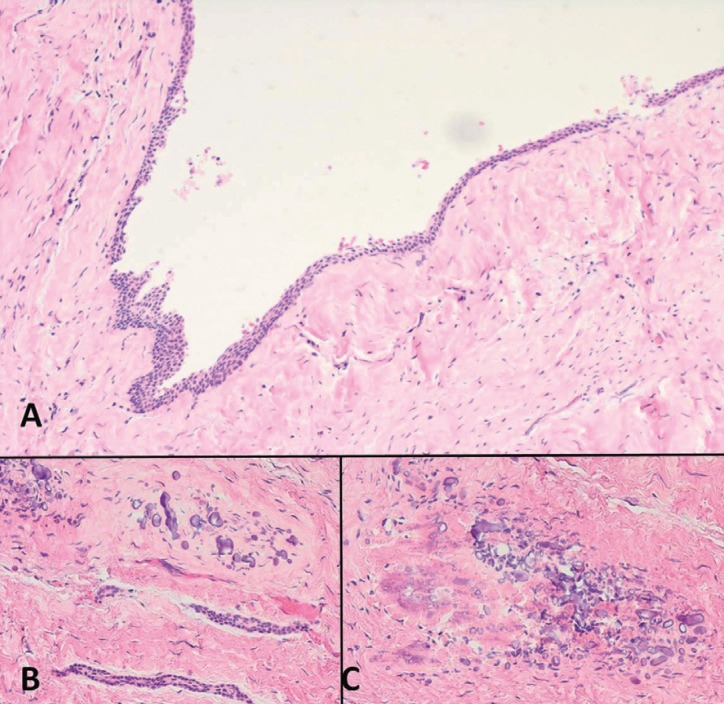
A) Cystic lesion with a fibrous connective wall without signs of inflammation and lined by a non-keratinized thin stratified epithelium (H&E 20x). B) Basophilic ovoid calcifications present at the connective wall and odontogenic epithelial cords (H&E 20x). C) Ovoid calcifications and peripheral eosinophilic areas with irregular disposition (H&E 40x).

Microscopically, a cystic wall constituted by fibrous connective tissue was observed. Interestingly, some basophilic globular calcified ovoid deposits were seen into its thickness. Furthermore, some of these had a greater size and presented a cemento-osseous structure with apposition lines and included cells. Some capsular deposits showed a non-keratinized stratified thin epithelial lining, focally detached (Fig. 2). The histopathological diagnosis was dentigerous cyst with capsular calcifications.

## Discussion

The present case report presents clinical and radiological characteristics that could be initially correlated with a benign odontogenic neoplasm. Generally, dentigerous cysts are asymptomatic unless they become secondarily infected or achieve a large size producing bone deformation or adjacent tooth retention or displacement (1,3). Lower third molars are the most affected ones by this pathology (2,6). A follicular space greater than 3 mm measured on the radiograph must be considered as a dentigerous cyst (4).

A thorough clinical and radiographic evaluation is needed for the diagnosis of pathology of the jaws. The final diagnosis is only obtained through the histopathological exam. Despite of the low frequency, some pericoronal lesions could correspond to benign tumors such as adenomatoid odontogenic tumor (7), calcifying epithelial odontogenic tumor (8) or even to malign tumors as the primary intraosseous squamous cell carcinoma or the central mucoepidermoid carcinoma (9). The histopathological diagnostic criteria for a dentigerous cyst includes the presence of a cystic cavity with a non-keratinized epithelial lining and a fibrous connective tissue wall (1). Some studies (10,11) specify that, histopathologically, there is no difference between an enlarged dental follicle and a small dentigerous cyst, although in a great majority of cases, the cyst is diagnosed in the presence of an epithelial lining (12). It is noteworthy that approximately half of dental follicles have some cystic degenerations and that the development of a real dentigerous cyst have a much lower incidence (12).

There are some published cases of dentigerous cysts containing calcified deposits that, radiographically, were provisionally wrongly diagnosed as benign odontogenic tumors with calcifications (13). This situation has also been described in another odontogenic cysts such as the residual cyst (14).

The differential diagnosis of a lesion that contains radiopaque material includes the calcifying cystic odontogenic tumor, ameloblastic fibrodentinoma, ameloblastic fibro-odontoma, central odontogenic fibroma, ossifying fibroma or odontoma (13). The presence of distrophic calcifications into dentigerous cysts is not excepcional, as stated by Lin *et al.* (1) in a study that evaluated 338 cases in which they found a 13.3% of them with calcified deposits. However, it is not described if the calcifications could be observed in the radiographic exam.

A retrospective study published by Shimizu *et al.* (13) analyzed radiographically 22 mixed lesions that included the crown of an included tooth and a 36.3% of them proved to be dentigerous cysts. An 87.5% were associated to included lower third molars, an 80% had thinned the surrounding bone, the 100% had resorbed the alveolar ridge, a 90% had bone sclerosis around and the 100% of intralesional calcifications were found near to the crown and the majority of them had less than 3 mm of size.

Calcifications on the cystic wall may constitute a degeneration of pericoronal connective tissue possibly related to its evolution. Nevertheless, its development remains unknown although they could be considered to be origined from dental follicle mesenchymal cells with the capacity of differentiate in cementoblasts or osteoblasts, producing a calcifying matrix (15). A study reporting hyperplastic dental follicles found a 40% of calcified deposits into the connective tissue but only a 6% showed radiopacities mostly associated to a greater time of follow-up (15). Besides, Sridevi *et al.* (14) published a case report of a long-term residual cyst that showed calcifications visible on the x-rays.

In our case, the dentigerous cyst and the development of cemento-osseous tissue was produced after a follow-up time of 10 years.

## Conclusions

This case report shows the relevance of performing a thorough clinical and radiological (2-D and 3-D imaging methods) exam of included third molars with pericoronal lesions to build a correct differential diagnosis.

Thus, non-neoplastic lesions such as dentigerous cysts could develop radiopacities inside the radiolucent pericoronal area.
